# The development of depressive symptomatology, burnout and lifestyle in the Czech population – in years 2014-2020

**DOI:** 10.1192/j.eurpsy.2021.401

**Published:** 2021-08-13

**Authors:** M. Vňuková, R. Ptáček

**Affiliations:** Psychiatry, First Faculty of Medicine Charles University in Prague and General Teaching Hospital, Prague, Czech Republic

**Keywords:** depressive symptomatology, burnout, lifestyle, SMBM

## Abstract

**Introduction:**

It is clear from the literature that depressive disorder is closely related to lifestyle, however the relationship between burnout and lifestyle remains unclear.

**Objectives:**

The aim of this study was to present a comprehensive overview of depressive symptoms, burnout and lifestyle over the years. Furthermore, this study looks at the relationship between burnout, depressive symptomatology and lifestyle and seeks to clarify the extent to which burnout can be explained by these variables.

**Methods:**

Data collection took place in three waves. The first data collection was in 2014 (October/November), the second in 2017 (March) and the third in 2020 (March). The STEM/MARK agency conducted the data collection and collected answers from a representative sample of respondents using the CAWI method - computer-assisted questioning. These respondents were selected from the European National Panel. Because the target group was adults (18-65 years), an online survey was chosen. Internet penetration in this target population is sufficient and it was not necessary to use a combination of methodologies.

**Results:**

All 3 data collections identically show that for the model explaining burnout statistically significant variables are: age, depression and fatigue during the day. Other variables related to healthy lifestyles did not reach statistical significance.

**Conclusions:**

Even though the variables regarding healthy lifestyles have not reached statistical significance, their importance should not be underestimated. Mental well-being is closely linked to physical health and therefore a holistic approach to health should be emphasized and the rate of burnout should be regularly monitored.

**Disclosure:**

No significant relationships.

